# On-Device Intelligence for Malfunction Detection of Water Pump Equipment in Agricultural Premises: Feasibility and Experimentation

**DOI:** 10.3390/s23020839

**Published:** 2023-01-11

**Authors:** Dimitrios Loukatos, Maria Kondoyanni, Gerasimos Alexopoulos, Chrysanthos Maraveas, Konstantinos G. Arvanitis

**Affiliations:** Department of Natural Resources Management and Agricultural Engineering, Agricultural University of Athens, 75 Iera Odos Str., 11855 Athens, Greece

**Keywords:** Internet of Things, edge intelligence, machine learning, artificial neural networks, smart sensing, predictive maintenance, fault detection, activity tracking, embedded systems, digital agriculture

## Abstract

The digital transformation of agriculture is a promising necessity for tackling the increasing nutritional needs on Earth and the degradation of natural resources. Toward this direction, the availability of innovative electronic components and of the accompanying software programs can be exploited to detect malfunctions in typical agricultural equipment, such as water pumps, thereby preventing potential failures and water and economic losses. In this context, this article highlights the steps for adding intelligence to sensors installed on pumps in order to intercept and deliver malfunction alerts, based on cheap in situ microcontrollers, sensors, and radios and easy-to-use software tools. This involves efficient data gathering, neural network model training, generation, optimization, and execution procedures, which are further facilitated by the deployment of an experimental platform for generating diverse disturbances of the water pump operation. The best-performing variant of the malfunction detection model can achieve an accuracy rate of about 93% based on the vibration data. The system being implemented follows the on-device intelligence approach that decentralizes processing and networking tasks, thereby aiming to simplify the installation process and reduce the overall costs. In addition to highlighting the necessary implementation variants and details, a characteristic set of evaluation results is also presented, as well as directions for future exploitation.

## 1. Introduction

The increasing nutritional needs of a continuously growing global population and the parallel depletion of natural resources pose serious challenges that need to be tackled successfully, especially in the agricultural sector. Water is the most precious among these natural resources and the dominant key element for agricultural productivity and food security, while worldwide agricultural irrigation accounts for 70% of freshwater [1]. Water pumps are crucial parts of the distribution network, as they are used primarily to move large amounts of water to the fields from their respective sources. These pumps are subjected to damage for a variety of reasons, such as inadequate water supply from the source, inefficient power, or a dirty water flow. To prevent the parts of the irrigation equipment from damage, it is important to monitor their working condition and take action when a malfunction is about to occur. Thankfully, the digital transformation of the agricultural sector seems to be a promising direction for solving the problems stated above [2], and this digitalization provides fertile ground for monitoring, automating, and analyzing agricultural operations via innovative approaches, including machine learning (ML), Internet of Things (IoT), and edge computing (EC) techniques.

The branch of artificial intelligence (AI) called ML is a prime technology to study. During the last years, the availability of vast amounts of data and information has allowed for more correct predictions to be made using ML models, while traditional machine learning algorithms are supported by methods that lead to the development of faster and more dynamic algorithms with better accuracy [3]. Machine learning models have the capability for learning and adapting according to the problem that needs to be solved, while conventional programming options are limited by the fact that the individuals who implement them are expected to already possess knowledge of the idiosyncrasies of the system the solution is being tailored for [4]. In this regard, the machine learning approach reduces the need for “experts” by introducing generally applicable and well-documented methods that can be followed easily by a wider number of scientists in the sector. The rapid expansion of available data due to the emergence of better sensor technologies has already increased the importance of machine learning and has made it a powerful tool for many applications across many disciplines. Consequently, machine learning can be a successful approach for detecting faults in a motor and diagnosing the type of malfunction that has occurred.

The IoT is a rapidly growing technology that involves devices being connected to the Internet, equipped with sensors, transducers, radio transceivers, and actuators, all working as a whole to gather, exchange, and respond to information [5]. In this regard, the IoT makes agricultural operations more efficient and fosters production [6]. Recent studies have highlighted the role of the IoT technologies in critical agricultural processes [7], including for precision farming, livestock monitoring, and greenhouse applications, with irrigation and water management activities drawing more attention [8,9]. Deterministically, as the world will be populated by billions of connected devices [10], the increased amount of data traffic that the networks will need to handle will be a serious cause of bottlenecks, generating latency, reliability, and privacy problems [11,12].

**Figure 1:**
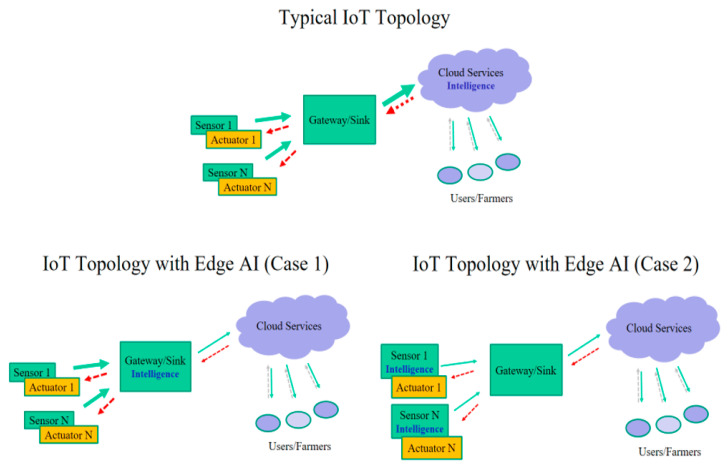
Network traffic reductions while migrating from cloud-based to edge AI solutions, with the intelligence capabilities to be ported on the gateway node (**case 1**) and the sensor nodes (**case 2**).

The utilization of edge computing (EC) techniques can improve the performance and overcome challenges involved with cloud-based approaches by providing in situ processing and storage capabilities [13]. Adding intelligence to the edges of the network infrastructure is commonly called edge AI. Deploying machine learning locally reduces the network traffic by allowing computations to be performed close to where the data are produced, preserving privacy when uploading data, in addition to reducing power consumption and networking delays that are traditionally required for continuous wireless transmission to remote gateways or to central cloud servers [12]. Indeed, there are two alternatives to edge AI implementations. According to the first one, the intelligence necessary for classification and decision-making migrates from the cloud to the gateway or sink node, which typically gathers the raw data from the peripheral sensor devices. This solution reduces the traffic toward the cloud and preserves privacy but still obliges the sensor nodes to deliver a considerable amount of unprocessed data to the local gateway node. Going further, the second alternative ports the intelligent operations on the peripheral sensing devices, thereby relaxing their network modules, extending their energy autonomy, and simplifying the node implementation requirements. The latter and more drastic case of edge AI is also known as “on-device” intelligence. Figure 1 provides a graphical explanation of the process of migrating intelligence from the cloud entities to the peripheral sensing nodes.

In the case of agriculture, ML is utilized primarily in applications that directly affect and improve agricultural processes. Some fundamental examples include crop management applications (61%), mostly for yield prediction, disease detection, weed detection, crop quality monitoring, and species recognition, whereas others involve livestock, soil, and water management aspects [14]. While such approaches comprise the majority, only a limited selection of ML-based applications focus on agricultural machinery and equipment, mainly for predictive maintenance. The latter field is of increased importance in order to avoid unpleasant conditions during the operation of farm machines. Various monitoring and control technologies and systems can be utilized. These systems aim to prevent the critical equipment from potential failures and damage, which otherwise can result in economic and production loss.

The area of predictive maintenance (PdM) has gained prominence, especially in the last few years, for various reasons. The condition monitoring of electric motors and other equipment used by industry avoids several economic losses resulting from unexpected failures and improves efficiency. More specifically, PdM is a technique that utilizes condition monitoring tools to monitor the performance of equipment during operation. Machine learning approaches seem to provide effective solutions in these areas, facilitated by the increasing capabilities of the hardware, cloud solutions, and advanced algorithms. The ML-based predictive maintenance techniques can be divided into two main categories, supervised and unsupervised. The supervised technique refers to the method where information on the occurrence of failures is present in the modeling dataset, while with the unsupervised technique process information is available but there are no maintenance data [15,16,17,18]. The availability of maintenance information mostly depends on the existing maintenance management policy. Supervised solutions are preferable when possible, although in the manufacturing environment sometimes it is required to develop a model with minimal or no historical research data, where unsupervised learning is a better option. Simple vibration data from an exhausted fan can be fit to different unsupervised learning algorithms and used to develop an ML model for fault detection [19]. In general terms, based on the literature review, the support vector machine (SVM), reinforcement learning (RF), and artificial neural network (ANN) algorithms are the most used machine learning algorithms for PdM, and most of the data used are real data, with only a few studies applying simulated or synthetic data while developing the ML models. Additionally, the most used data are vibration signals acquired by accelerometers, and the most applied ML category is classification [20]. Furthermore, in some applications, the integration of ML-based PdM leads to positive results, reduced costs, and improved efficiency and safety, while there is no need for equipment replacement [21].

A handful of analogous studies have been conducted to detect malfunctions using machine learning techniques in water pumps. Some of the methods of analyzing failures in water pumps include the use of low-cost accelerometers paired with machine learning classifications [22] and techniques used to forecast water demands [23]. In a parallel manner, machine learning models have been implemented for the early fault prediction of industrial centrifugal oil and gas pumps [24]. Unfortunately, most of the research on smart agricultural applications is mainly focused on cloud-based solutions rather than on-device deployment, and only a few scholars have applied combinations of cloud, fog, and edge computing techniques in the agricultural domain [25].

Based on previous experiences in the field of on-device ML deployment, such as the development of a classification model used in a commercial faucet to determine and alert the user about water consumption [26], this paper highlights the feasibility of developing a low-cost prototype system, exploiting properly trained models in order to classify and diagnose potential motor faults of a centrifugal water pump. The research being conducted indicates that by using low-cost sensor data, such as from vibrations intercepted by a three-axis accelerometer, an encompassing and accurate view of the conditions affecting the system can be provided.

The priority was to provide a model capable of intercepting and classifying the different operating conditions of the pump with satisfactory accuracy. Particular attention was also given to simplifying the installation process of the monitoring mechanism on the pump and making it as less invasive as possible for the hosting system. The latter arrangements are translated into minimized wiring and bolting requirements, without the need to cut the piping or the power wires to add extra sensors. The whole study aims to deliver a compact, low-cost, on-device intelligent solution for malfunction detection that drastically reduces the dependence on external cloud resources and minimizes their privacy, cost, and complexity disadvantages.

The discussed approach aims to highlight the feasibility of implementing fluently working solutions using very limited hardware resources and suitable software tools. More specifically, it utilizes recently emerged platforms facilitating the training of the underlying neural network (NN) models and their adaptation so as to be executed, in situ, by innovative yet inexpensive microcontrollers with increased memory and processing capabilities, as well as well-known radio transceivers. The accuracy of the NN model is satisfactory (at nearly 93%) and can be further improved by using more extensive training and sampling variants. Finally, a wider perspective is provided, as diverse implementation arrangements for the sensing, decision, and communication parts were investigated and assessed.

Apart from this introductory section, the rest of this paper is organized as follows. Section 2 highlights the motives and preparatory arrangements behind this work. In Section 3, interesting hardware and software implementation details are given. Section 4 provides a multi-perspective performance evaluation of the proposed malfunction detection system and discusses its strengths and weaknesses. Finally, Section 5 contains some important concluding remarks and directions for future investigations.

## 2. Methodology

This section provides further insight into the motives in order to keep agricultural equipment, such as water pumps, in good condition, as well as insight into the recent technological advances that facilitate the methods used to detect malfunctions at reduced cost levels. Furthermore, the necessary functionality requirements and the interoperation and component selection details are presented for the delivery of a fluent system capable of distinguishing among the diverse operating conditions (and potential malfunctions) of a typical centrifugal water pump and of reporting the corresponding alerts to the end user. Special attention is paid to highlighting the feasibility and implementation steps of the fluent ML solution by utilizing minimal hardware resources and properly tailored software.

### 2.1. Motivations and Challenges

Mechanization is a crucial input for the agricultural sector. Factors that reduce the availability of farming power resources, such as mechanical equipment malfunctions, compromise the ability to cultivate sufficient land areas and have long been associated with production and profit declines. The use of sustainable agricultural mechanization techniques can also contribute significantly to developing value chains and food systems more efficiently and effectively, in an environmentally friendly manner. The implementation of increasing levels of mechanization does not necessarily involve sizable investments in tractors and other machinery. The producers should resort to power sources that best fit each operation, depending on the tasks to be done and the individual performing them [27]. They should also work on preserving their existing equipment and acting whenever a malfunction occurs. Preventing permanent damage to farm machinery (i.e., tractors, irrigation systems, pumps) is imperative, as this reduces economic losses and contributes to sustainable development.

Pumps are key elements in any irrigation system, and taking into account the hydroponic, aquaculture, and even symbiotic perspectives of modern agricultural ecosystems [28], their role is further extended beyond covering the conventional water needs of the plants. In this regard, water pumps can be used to carry dissolved nutrition ingredients for fishes or fertilizers for plants. Furthermore, the scarcity of the water resources, in conjunction with the large quantities required in a typical agricultural premise, often make water recycling a necessity. These exemplary operations are translated into additional functions for the pumps, which usually have to tackle the disturbances in water flow due to the presence of mud or membranes and filters. Desalination systems, used for either plant or animal wellbeing, also have similar requirements [29]. When extending the piping network or performing spraying with fertilizer solutions, stenosis phenomena can be observed (e.g., to the nozzles), resulting in increased pressure that makes the pumps underperform or become damaged. Pumps can not only be installed at fixed locations but also as part of autonomous robotic spraying systems [30]. Even under more conventional settings, the water supply can also suffer due to broken or bent pipes (e.g., damaged by tractors or animals) or wells containing marginal water quantities. All of these reasons make the fluent operation of the water pumps a challenging situation and the awareness of any potential malfunction a high priority.

Smart agriculture refers to the use of advanced technologies, including big data, the Internet of Things, cloud resources, and fog and edge computing, as well as essential tools for tracking, monitoring, and automating agricultural operations [31]. Toward the successful digital transformation of agriculture, the utilization of innovative technologies seems to be a key factor in addressing issues related to agricultural applications. The rapid development of the electronics industry has assisted in the increase in the quantity and quality of several components, such as microcontroller units (MCUs), single-board computers, sensors, and radio transceivers, at affordable cost levels. Specifically, the new generation of microcontrollers, apart from arranging typical sensing and acting tasks, can support complex operations with reduced execution times, as they have faster and more efficient processors and more memory. Additionally, the advanced contemporary radio technology is capable of long-range transmission with lower energy consumption rates.

These advancements in smart agriculture have paved the way for the employment of machine learning, particularly using artificial neural networks (ANNs), in a broader assortment of applications and projects, while having the benefit of being simplistic enough to be implemented and deployed even by non-expert individuals. This ease of deployment is assisted by the notion that these ML algorithms run on the end devices of a given system; for example, on the hardware of the sensor node. The aforementioned devices are miniature in size and are equipped with energy-saving or -harvesting options that extend their operation, while their processing capability and memory capacity are improved due to the mentioned advancements. These features combined allow for the local execution of ML algorithms, with drastically minimized energy and networking footprints and noticeable cost-effectiveness compared to the typical central system approach.

Another reason that favors the applicability of ANNs on these microcontroller units (MCUs) is the progress in the corresponding training, programming, and deployment tools and software platforms that exhibit increased user-friendliness. It must be noted that the training phase of the artificial neural networks remains significantly more computationally demanding than the execution phase. This is because during the training process a significant amount of sampled data are processed to prepare the model and make it accustomed to the parameters of the problem. Once the ANN is trained, the model can be used repeatedly for inference actions without requiring as much computational power, which is optimal for MCUs with fewer resources. Despite this, several of the trained models can potentially still be heavy for these MCUs. Thankfully, it is possible to create trimmed-down versions of these more complex models that are compatible with the improved generation of MCUs [32], using user-friendly tools such as TensorFlow Lite [33], in the context of TinyML [34].

### 2.2. Functionality Overview and Component Selection

Benefitting from the dynamic presented in Section 2.1, this study utilized machine learning to develop a classification model that can be executed locally on the sensing nodes to detect characteristic malfunction cases of a typical water pump that is part of a standard irrigation network. This was an attempt to further explore the implementation of ML in the engineering field and to develop smart agriculture solutions, which are referred to as the future of the digitalization of farming.

Some common types of water pump malfunctions include air intake and stenosis issues in the inlet or outlet of the pump, which occur for a variety of reasons, from mechanical deformation of the pipes to mud concentration inside them. In this regard, in order to achieve successful diagnoses of the most typical problems in the operation of pumps, characteristic malfunction scenarios were generated by placing three valves that intervene between the plastic tubes and the pump. More specifically, via suitable valve readjustments, the three most probable cases of potential malfunction scenarios were emulated as external disturbances and compared against the normal operation of the water pump. In addition, for more accurate training, a fifth class of data was included, corresponding to the pump motor being switched off to emulate cases of noise. Sensor data were recorded for all of these classes. Various data source types were evaluated (e.g., acoustic, current, motion) with the accelerometric ones to provide better results. In turn, a dataset consisting of five different classes was created and used to train the ML model.

The necessary hardware components were an AC centrifugal water pump, part of which was retired equipment, and a 50-liter water tank mounted on a custom base, connected to the water pump using 3/4 plastic hydraulic tubes with plastic valves intervening between the piping. Additionally, this approach uses off-the-shelf hardware modules that are easy to find, well-documented, and cost-effective. Firstly, an Arduino Nano 33 BLE Sense [35] board was used, which is a microcontroller board with a powerful processor that offers the ability to develop larger programs compared to those of an Arduino Uno, as it has flash memory that is 32 times bigger and RAM that is 128 times bigger. The advanced features of the Arduino Nano 33 BLE Sense board make it suitable for supporting the processing and memory-demanding tasks that are required for executing a trained machine learning model. The suitability of the Arduino Nano 33 BLE Sense board for supporting similar tasks was indicated by other on-device intelligence projects [36]. In addition to the Arduino microcontroller, a NodeMCU module, based on the ESP8266 [37] chip, was utilized, offering a variety of WiFi network connectivity options.

The Arduino Nano 33 BLE Sense unit had to be programmed for the following functions:

- To record and transmit sensor data, mainly motion data, through its built-in sensors (e.g., accelerometer);
- To forward these data, assisted by a more powerful computer, to the cloud, in order for the suitable neural network model to be trained;
- To execute the trimmed-down neural network model, i.e., to identify the operation state of a pump, and to communicate its decision to the network.

For the machine learning part, the training and incorporation of the final artificial neural network (ANN) [38] model into the software running on the microcontroller is required. In particular, an ANN is based on the operation of neurons of the human brain. This structure has one input layer and one or more hidden layers that are interconnected, as well as an output layer to deliver the results. A simple and efficient development platform for creating machine learning models (i.e., to train and extract or compile data) on edge devices is the Edge Impulse cloud environment [39]. This platform supports plenty of development boards, including the Arduino Nano 33 BLE Sense unit. This allowed for the instant recording, uploading, and labeling of the samples required for the dataset and the direct deployment of the final model’s software. Further requirements were addressed via the Arduino IDE [40] programming environment.

**Figure 2:**
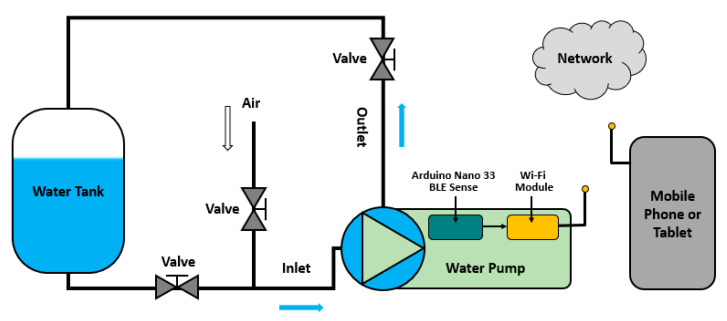
A functionality overview of the proposed water pump malfunction detection system.

A network-based monitoring environment was also developed to inform the user of the operating conditions of either one or more pumps. The presence of a sink or gateway node nearby for gathering the information from the sensor nodes, especially in cases where more than one water pump units should be monitored, facilitates the connectivity with the end-user device (e.g., smart phone, tablet, or laptop) and also makes the classification decision information available to the cloud for easier access and further visualization or processing. The details for the latter arrangements are beyond the focus of this research work. Figure 2 provides a functionality overview of the proposed water pump malfunction detection system.

It must be noted that apart from the performance of the ANN model, an important issue to be examined was the applicability of a satisfactory solution with minimal costs and complexity, using on-device machine learning techniques, having a drastically lower network load, and achieving better communication distances and a smaller energy footprint.

The adoption of the Arduino Nano 33 BLE Sense unit accompanied by a WiFi radio was not the only possible implementation arrangement. Actually, it remains the best option for communicating the technological principles behind this study, but it is not optimal in terms of the cost and radio coverage. For this reason, this work goes further by providing alternative implementations utilizing the recently emerged and cheaper RP2040 microcontroller, as well as transceivers with improved efficiency.

## 3. Implementation Details

In accordance with the component selection and functionality directions provided in Section 2.2, Section 3 presents characteristic details of the implementation process. More specifically, the basic construction of the system as well as the initial setup and preparation process are highlighted, in order to gather data for the training process and to add machine learning capabilities to the whole system. The details for the training of the ANN model, the incorporation of the trained model into the microcontroller of the water pump fault detection system, and the communication arrangements are also explained. Additionally, the observed alternative hardware implementations and extensions are also mentioned.

### 3.1. Description of the Basic Construction

The system was designed to be of the closed type, which means that the water flows from the 50-liter tank to the water pump and ended up back in the tank to be recycled. Thus, both the water pump’s inlet and outlet were connected to the water tank with plastic tubes, with intervening valves to control the water flow in each tube. In addition to the valve, another tube was perpendicularly attached to the tube connected to the pump’s inlet. This tube was also equipped with a valve; however, the other end of the tube was left open to allow the influx of air, depending on the valve’s current state.

**Figure 3:**
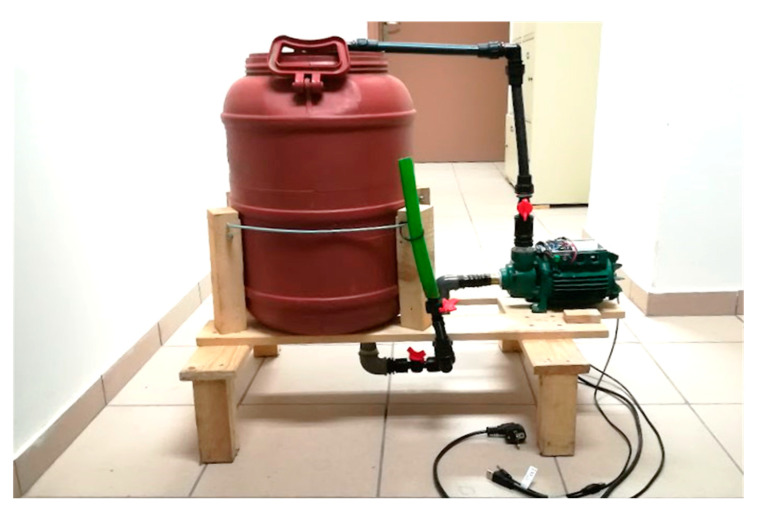
The implementation details for the proposed system, including a centrifugal water pump and three intervening plastic valves in the piping.

The Arduino Nano 33 BLE Sense unit, along with its in-built sensors (e.g., microphone, accelerometer), was fixed on the side of the water pump motor to sense the different vibration patterns for each data class. For the data acquisition, it is required to connect the Arduino Nano to a computer through its USB port to easily record and upload samples to the specified Edge Impulse project. The same connection can be utilized to compile and upload the trained model to the Arduino Nano BLE and to monitor the model’s performance. Finally, the Arduino Nano BLE was wired to the NodeMCU unit to allow access to the WiFi network. Figure 3 provides implementation details of the proposed system, including a centrifugal water pump and three intervening plastic valves in the piping.

### 3.2. Setup and Preparation

As mentioned above, the Arduino Nano 33 BLE Sense unit is fully compatible with the Edge Impulse platform, and they can easily collaborate as long as the device is set up in Edge Impulse. In order to successfully connect the board, Arduino CLI and the latest Edge Impulse Firmware were downloaded. The board was connected to a computer through a USB port, and the reset button was pressed twice in quick succession to launch the bootloader. Next, the flash script for the suitable operating system was opened (in this case flash_windows.bat) to flash the firmware to the board automatically, without involving the user interface, then finally the device was ready to connect to the Edge Impulse platform. In order to easily relay data from the in-build accelerometer of the Arduino Nano 33 BLE Sense unit to the Edge Impulse platform over a serial connection, the data forwarder tool was used through a Raspberry Pi 4 Model B board as an assistive computer unit.

### 3.3. Neural Network Training

**Figure 4:**
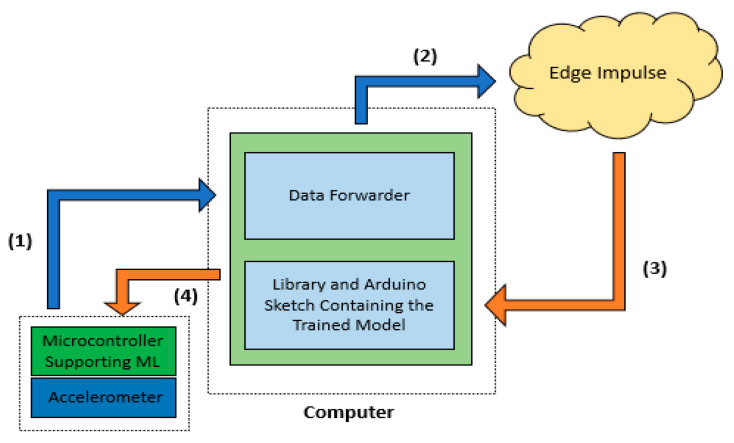
The necessary sequence of steps to be followed for the machine learning training and deployment processes: sensor data acquisition (1), data preparation (2), model design and creation (3), optimized model deployment onto the in-situ microcontroller (4).

The basic steps of the machine learning training process are depicted in Figure 4. The first step in training a neural network model is to acquire a sufficient amount of data for each particular class. This dataset consisted of five classes, one for normal operation data, three for the simulated malfunction data, and a final one for data considered as noise. A sample length of five minutes or more is adequate for such a project, as the purpose was predominantly experimental. The collected data then needs to be split between a training dataset, which is used to train the neural network, and a testing dataset, which is reserved for testing the efficiency of the model. Since all collected data are automatically uploaded to the training set, it is suggested by the platform developers that about 20% of the data be allocated to the testing dataset. However, the percentage may vary slightly as the total amount of collected samples may not be divisible in such a way, as was the case in this project, so the split was performed over a range of 78–22%, which did not affect the model’s performance or efficiency.

After collecting and splitting the necessary data, the next step is to design and train the model, which requires the addition of a processing block that modifies the data and a learning block that essentially allows the selection of the specific neural network to be trained. The data were mainly collected with the Arduino Nano unit’s built-in accelerometer, so the proper block for processing such data had to be selected, which was the “spectral analysis” block, since it is essential for analyzing repetitive motion, such as data from accelerometers, and extracting the frequency and power characteristics of a signal over time. For the learning block, a Keras-implemented classification neural network library was utilized, which can learn patterns from the given data and apply these to new data. Such a library is fitting for categorizing movements or recognizing audio, the former being the main objective of this experiment. Furthermore, the window size was set to 2000 ms (i.e., 2 s), according to the profiles fed into the training system and considering the period of the phenomenon. In a similar pattern, the window increase was set to 80 ms and the frequency to 100 Hz.

The processing block generated 33 features, which were imported as the input layer of the training process. The intermediate layers included 10 and 5 neurons accordingly, the number of training cycles was set to 30, and the output layer contained the 5 classes. After the training process, Edge Impulse saves the model with the best performance, in the Quantized (int8) version, which is suitable for the Arduino hardware platform. Edge Impulse gives the option to download the model as a code (library and sketches) for Arduino, accessible via the Arduino IDE environment, and run it in real-time.

### 3.4. On-Device Model Integration and Communication Arrangements

**Figure 5:**
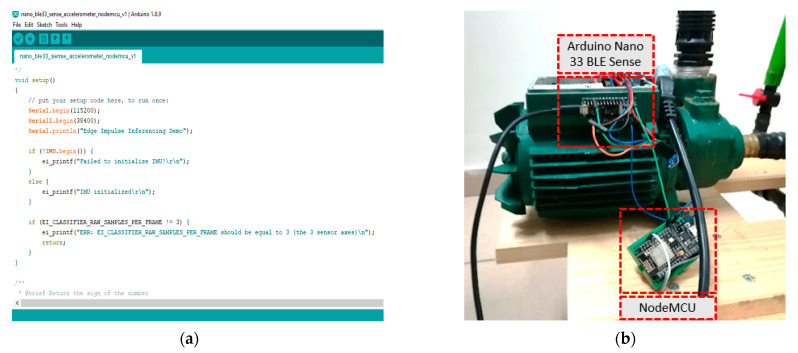
(**a**) The Arduino IDE environment utilized for porting the trained neural network model onto the microcontroller and further code customization options. (**b**) Details of the 2-MCU design of the on-device intelligence solution utilizing an Arduino Nano 33 BLE Sense unit and a NodeMCU board.

The code generated by the Edge Impulse platform, in the form of a generic Arduino library, provides customizable examples (sketches) for the Arduino IDE environment, with the Arduino Nano 33 BLE Sense board being among the supported models, making it compatible with the generated model parameters. The selection of the “Arduino library” option provides freedom in combining the machine learning model with additional algorithmic behaviors necessary to be executed by the hosting microcontroller. Further customization and the final porting process onto the microcontroller board were possible via the Arduino IDE environment. Figure 5a depicts an instance of the latter environment during the programming process. Appendix A provides further implementation details for the software running on the pump.

Further arrangements were made in order for the pump status predictions to be seen by the user in real time. An obvious option was to use the USB interface of the Arduino Nano 33 BLE Sense unit and a terminal application, such as the serial monitoring component of the Arduino IDE environment, running on a computer. This option is fine for programming and debugging purposes but remains of limited practical importance for users who prefer wireless access to the information being generated. Unfortunately, the Arduino Nano 33 BLE Sense unit provides a rich set of diverse sensors (i.e., acoustic, luminosity, color, temperature, humidity, barometric, motion, and magnetic), which make it ideal for prototyping investigations but it lacks a WiFi radio module. As the latter is the standard for everyday radio communication, a second board, based on the ESP8266 chip, namely a NodeMCU unit, had to be utilized. More specifically, the NodeMCU WiFi-capable module was connected to the Arduino Nano 33 BLE Sense through one of the serial ports of the microcontroller. Only the serial TX pin of the Arduino had to be wired with the RX pin of the NodeMCU, plus the common ground pin. If both devices are not using their USB connection plugs for communicating with a computer (which was frequently done during the experiments for debugging purposes), the power supply pins of the Arduino Nano 33 BLE Sense and the NodeMCU should also be wired in order for both devices to function, provided that one of them is properly powered. The programming of the NodeMCU was also performed via the Arduino IDE software. Details of the interconnection between the Arduino Nano 33 BLE Sense and the NodeMCU board, during the tests on the pump, are shown in Figure 5b.

The combination of the trained ANN model implementation with simple and more conventional programming techniques improved the prediction accuracy, as in the case of the air intake category. This arrangement was favored by the presence of a second microcontroller unit. More specifically, statistical post-processing was applied to the original ANN decisions after their arrival from the Arduino Nano unit to the NodeMCU board. The latter board calculated the most probable category over a fixed period of consecutive samples, prior to finalizing the result, without modifying the initial neural network model being trained. The final results were then available to the user via the TCP/IP connectivity options of the NodeMCU board. The option to suppress consecutive notification messages of the same type was also implemented on the NodeMCU board.

In greater detail, the NodeMCU unit, utilizing the ESP8266WiFi library [41] for the Arduino IDE environment, was initially set up as a web server hosting a simple HTML page with dynamic content corresponding the operational condition of the water pump. Further arrangements allowing the NodeMCU board to deliver its characterization decisions to a Raspberry Pi 3 Model B sink device [42] were made for permanent storage and better visualization. The role of the sink and gateway node software can range from being comparatively simple to quite complicated, depending on the degree of functionality and level of user friendliness. With the simpler approach, programmed in the Python language, the data collected from each pump are written into files, and these files become publicly accessible via a locally running web server, such as with Apache2 software. More sophisticated solutions, including experiments with well-known dashboards and IoT platforms, were also possible but were beyond the objectives of this study.

### 3.5. Alternative Hardware Implementations and Extensions

Apart from using acceleration data to intercept pump operation problems, acoustic data, provided by the Arduino Nano 33 BLE microphone, and current consumption data, provided by an ACS712 Hall sensor installed in series with the motor of the pump, were also utilized as potential classification data sources in conjunction with the Edge Impulse platform. Nevertheless, both techniques exhibited lower accuracy compared with their accelerometric data counterparts.

The selection of the Arduino Nano 33 BLE Sense unit was a “safe” choice for efficiently training the water usage classification (characterization) model, but the experimentation with more optimized or standardized hardware components, compared with the Arduino Nano 33 BLE Sense unit, was a significant challenge. Indeed, the latter microcontroller has plenty of sensors that are very appropriate for general purpose experimentation but also increase the cost of the overall implementation. Furthermore, the deployment and testing process in open-field environments using long-range radio networks such as LoRa was a priority that could not be overlooked. Both cases were examined in this research work, as the necessary “fresh” and suitable tools and methods were available.

**Figure 6:**
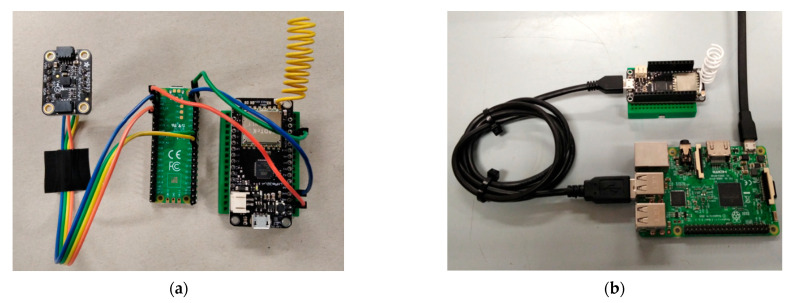
(**a**) Alternative on-device intelligence arrangements involving an external accelerometer, a Raspberry Pico, and a LoRa32u4 radio module, from left to right, respectively. (**b**) Gateway and sink node implementation using Raspberry Pi 3 Model B and a LoRa radio.

In this regard, the Raspberry Pi Pico [43] microcontroller board was also a participating candidate in the deployment process, which apart from its very attractive price, has sufficient processing power and memory size (due to its RP2040 chip) to meet the requirements of the underlying tasks, and also offers many standard connectivity and power options. The lack of a built-in accelerometer makes the use of a motion sensor pairing the module on the Arduino Nano 33 BLE Sense unit necessary, such as the Adafruit LSM6DS33 unit [44]. Similarly, the lack of a radio interface (preferably suitable for low-rate, long-range communication) is counterbalanced by the adoption of a cheap AVR microcontroller board, running at 8 MHz and equipped with a LoRa radio, namely a LoRa32u4 unit [45]. The latter unit is also capable of performing the additional tasks the NodeMCU board does, such as the necessary data post-processing functions, in a similar way to the one explained in Section 3.5. Figure 6a depicts the alternative arrangements involving an external accelerometer, a Raspberry Pico, and a LoRa32u4 radio module, from left to right, respectively. A LoRa32u4 module was also fitted via a USB port on a Raspberry Pi 3 Model B unit, in order for the latter to act as a sink or gateway for the malfunction notifications from the pump(s) towards the end user. Figure 6b depicts the gateway setup in order to support LoRa radio communication with the in situ equipment. For denser radio traffic scenarios (e.g., for many pumps in operation and additional water flow data delivery), a LoRa concentrator board, fixed on the Raspberry Pi unit, would be a more efficient solution.

The presence of an electric water pump facilitates the powering of the malfunction detection system, which can be done using a 5V DC power supply. Nevertheless, a pair of Li-ion batteries, e.g., 18650 batteries, connected to a charger guaranties a more stable operation.

The scenario of complete independence from the power system of the water pump is also possible, as the overall system has low power consumption, supports sleep mode, and can be woken up by an external electrical signal (e.g., by the starting of the operation of the water pump) or periodically by a timer, as notifications do not have to be delivered continuously. The LoRa32u4 unit can drive the sleep/wake activity pattern for the overall system, as it is very efficient in its low-power operation, and the rest of the equipment can be powered by its GPIO pins and cheap voltage-boosting component. The microcontroller–radio transmitter system can also be used to stop the motor at the remote user’s request or automatically based on malfunction detection. In order for these tasks to be performed, a power relay with a suitable rating was utilized.

## 4. Results and Evaluation

This work emphasizes the detection of faults in a water pump using an ML-based predictive maintenance technique with sufficient accuracy, applied on the Edge Impulse platform, which reduces the amount of information that needs to travel and leads to improved privacy and decreased communication loads and energy consumption. The adoption of simple, long-range, and low-energy radios facilitates the whole process. More details regarding the results and the technical evaluation are given in Section 4.1, Section 4.2 and Section 4.3.

### 4.1. Neural Network Model Performance

For classification evaluation algorithms, accuracy is the most frequently used indicator for either binary or multi-class classifications problems, as well as a statistical table called the confusion matrix. In predictive analytics, a confusion matrix is a two-dimensional matrix, with two rows and two columns that report the numbers of true positives (TP), false negatives (FN), false positives (FP), and true negatives (TN) [46,47,48,49,50]. In Table 1, the columns represent the actual classes while the rows represent the predicted classes [51,52,53].

For more aggregate characterization applications, the accuracy metric is used to express the degree to which the predictions of a model match the model’s reality, and is usually given as a percentage. In general, the accuracy metric measures the ratio of correct predictions over the total number of samples evaluated:

(1) $$\mathrm{accuracy}=\frac{\mathrm{TP}+\mathrm{TN}}{\mathrm{TP}+\mathrm{FP}+\mathrm{FN}+\mathrm{TN}}$$

**Figure 7:**
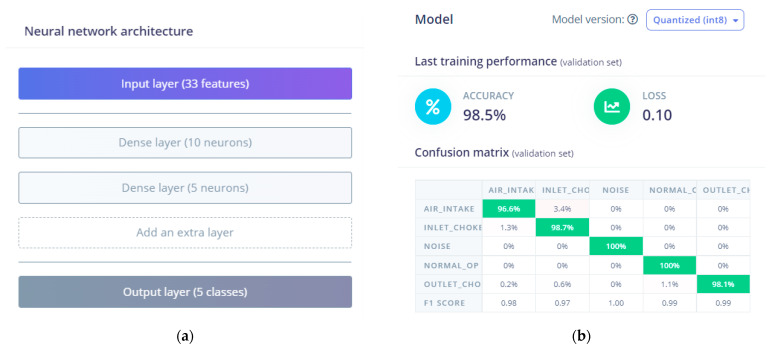
(**a**) The ANN model structure and (**b**) expected accuracy using the Edge Impulse platform.

In accordance with Section 3.3, a model structure providing a good compromise between the memory size requirements and accuracy contains one input layer, two hidden layers containing 10 and 5 neurons, and the output layer (Figure 7a). In the Edge Impulse platform, after the training process, the confusion matrix of the model was generated automatically and the overall percentage accuracy was calculated afterwards, which in this experiment was 98.5% (Figure 7b).

Additionally, the system generates the model’s testing accuracy, using data kept aside for this purpose. The system generated the right outcome for the normal operation category with 100% accuracy. Similarly, the air intake category achieved 97.2% accuracy, the inlet choke category 99.8% accuracy, and the outlet choke 96.7% accuracy, according to the Edge Impulse cloud environment. These performance results amassed score of a 98.71% total model accuracy using the testing data set. Additionally, a second evaluation stage was performed, which corresponded to the testing of the real system. Table 2 shows the confusion matrix after classifying 1056 episodes, more specifically a total number of 205 samples during the normal operation, 203 samples during the emulation of the outlet choke state, 208 during the emulation of the inlet choke state, and 230 throughout the air intake scenario.

Apart from the accuracy, some common performance evaluators are the sensitivity, specificity, precision, f-measure, and g-mean:

Sensitivity (or recall): The measures how much a classifier can recognize positive samples:

(2) $$\mathrm{sensitivity}=\frac{\mathrm{TP}}{\mathrm{TP}+\mathrm{FN}}$$

Specificity: This measures how much a classifier can recognize negative samples:

(3) $$\mathrm{specificity}=\frac{\mathrm{TN}}{\mathrm{TN}+\mathrm{FP}}$$

Precision: This is the ratio of predicted positive examples over the total predictive patterns in a positive class:

(4) $$\mathrm{precision}=\frac{\mathrm{TP}}{\mathrm{TP}+\mathrm{FP}}$$

F-measure: This is the harmonic mean of the sensitivity and precision:

(5) $$\mathrm{F-measure}=2\times \frac{\mathrm{precision}\;\times \;\mathrm{recall}}{\mathrm{precision}+\mathrm{recall}}$$

G-mean1: This is the geometric mean of the sensitivity and precision:

(6) $$\mathrm{G-mean}1=\sqrt{\mathrm{sensitivity}\;\times \mathrm{precision}}$$

The processing of the data being collected revealed that the total actual accuracy was 93.02% after testing the model with user-generated malfunctions using the valves being integrated into the system’s construction. It is worth mentioning that the model could recognize undesirable inlet choke cases, achieving 97.12% precision, while the air intake cases were predicted with 90% precision, the outlet choke cases with 85.71% precision, and the normal operation cases with 92.68% precision. This performance was close to the expected performance according to the testing of the model. On the other hand, there were some incorrect predictions, such as some cases where the model classified an outlet choke scenario as an air intake or normal scenario. These failures can be attributed to the fact that there was a small area where the borders of those categories overlapped, thereby confusing the neural network classifier. Furthermore, it should be noted that an additional 0.4 certainty threshold was programmed on the microcontroller for more reliable characterization. Additionally, in order to provide a more comprehensive understanding of the effectiveness of the model, some additional performance metrics described above were calculated and the results are depicted in Table 3. It can be clearly observed that the g-mean is high in most classes, which indicates that the model did not overfit negative classes nor underfit positive classes [53].

The NN performance measurements mentioned above are in accordance with the findings of other studies utilizing ML techniques for the classification or detection of malfunctions in pump equipment [17,18,22,23] or even the findings of more general studies, such as the results presented in [19,20,21].

### 4.2. Networking, Power Consumption, and Cost Issues

Several tests were performed to assess the network and energy footprint of the system variants being presented, following the techniques described in [54]. The corresponding measurements being collected were in-line with the results of other studies utilizing similar hardware and software arrangements [26,36] and with the datasheets from the component manufacturers [35,37,43,45].

In the case of the WiFi radio, the direct range coverage between the smart (i.e., the malfunction detection node) sensor and the gateway (or the user equipment) was almost 100 m, while for the LoRa radio this range was extended easily to 1 km. The overall power consumption of the system, including the accelerometer sensor, the Raspberry Pi Pico, and the LoRa 32u4 module, was around 40 mA during the activity and dropped to approximately 0.2 mA during the sleep period. The data transmission actions typically required an additional amount of nearly 80 mA for moderate power transmission settings and lasted for 50–60 ms. For the initial system setup, comprising the Arduino Nano 33 BLE Sense unit and the WiFi radio module, the total active-state consumption rate was almost of the same level (i.e., with the Arduino Nano 33 BLE Sense unit consuming 3–4 mA less than the Raspberry Pi Pico unit and the NodeMCU unit consuming 6–7 mA more than the LoRa32u4 module). The latter numbers could be further increased, as the WiFi scan and the transmit actions of the ESP8266 chip (inside the NodeMCU unit) could exceed the 100 mA level.

The major advantage of the proposed on-device system is that it has to transmit only the results of the classification and not the analytical raw data being measured, thereby saving a lot of energy and bandwidth. For instance, if the sensor, running at 100 sps, had to continuously transmit 3-axis accelerometric data at 2 bytes each, an approximate data rate of 100 × 3 × 2 = 600 Bps or 4800 bps would be required. On the contrary, as only the pump operation characterization decision (that is, taken after 2–3 seconds of recorded data) has to be transmitted, a few bytes every 2–3 seconds is enough. The suppression of repetitive decisions is also possible, further reducing this rate. Taking into account that the packet transmission actions are far more power-demanding than the processing actions (for the common microcontrollers utilized in this research), the drastic reduction in energy consumption is fully justified. Furthermore, the MCUs being used are of low power as well, while the sleep–wake or energy-harvesting methods can also be applied.

The total cost of each of the discussed malfunction detection equipment, after adding the 40 EUR for the Arduino Nano 33 BLE Sense unit, the 7 EUR for the NodeMCU unit, the 8 EUR for LiPo batteries, and the 5 EUR needed for a good-quality plastic enclosure box, was around 60 EUR. In a more commercial version, this value was further reduced by replacing the Arduino Nano 33 BLE Sense unit with the Raspberry Pico unit, which costs only 6 EUR. For this version, an external accelerometer such as the Adafruit LSM6DS33 unit had to be attached, costing 8 EUR. The replacement of the WiFi radio (via the NodeMCU unit) with a LoRa transceiver added 10 EUR to the total cost but saved energy and offered increased distance coverage.

The decision to use the LoRa32u4 board added an extra cost (of about 5 EUR, compared with a bare LoRa chip) but provided much better flexibility and power management options. The gateway node required 45 EUR for the Raspberry Pi 3 Model B unit, 15 EUR for the LoRa32u4 board, 5 EUR for the plastic enclosure box, and 5 EUR for the power supply. In conclusion, the cost of the latter gateway node was around 70 EUR.

### 4.3. Further Discussion

In this paper, several innovative technologies, software programs, and low-cost electronic components were exploited to detect malfunctions on a water pump for agricultural premises.

The ability to utilize diverse microcontrollers for ML actions at the edge, and more specifically on-device, was highlighted, a functionality that up until recently could not be supported efficiently. This behavior, in turn, resulted in a drastic reduction in the network traffic load towards the central (cloud) entities and apparently decreased their processing load, as only the classification or decision data had to travel on the network instead of the detailed raw data sequences containing sensor readings. The power consumption and data privacy, which benefited from this situation, were also improved, as processing is less power-consuming than packet transmission actions and little to no detailed user-sensitive data, e.g., utilizing cellular infrastructures, have to travel from the farmer’s premises toward the distant cloud.

The experiments that were performed indicated that the type or model of the sensor being used drastically effects the quality of the gathered data. For this reason, it is recommended to use the same device for both the neural network training stage and the real field classification application. The objective of this research was for the classification algorithm to be able to distinguish between as many malfunction cases as possible.

The limitations also had to be considered during the training, in terms of the sampling frequency and sampling duration of the sound data, due to the limited memory of the Arduino Nano 33 BLE Sense unit, as well as the data rate threshold between the microcontroller and the Edge Impulse cloud. The use of instantaneous current measuring techniques via the hall sensor was also constrained by these factors, and could possibly deliver more promising results at higher frequencies than the 100 sps that was achieved.

The overall cost, the complexity of the installation process, the complexity of the training process, the number of malfunction cases to be captured, the degree of invasiveness, the modifications required to the original system under monitoring, and the accuracy of the model were important factors to be taken into consideration.

The accelerometry-based methods being tested performed better than the acoustic and current-measuring ones but exhibited a dependence on the way the motion sensors were installed on the pump and the way the pump and its piping were fixed on the undercarriage construction. This makes the provision of datasets related to specific models of water pumps necessary and means these datasets must be complemented with additional in situ data, which should be addressed in the future.

The fact that some malfunction cases, such as the air intake class, contain hidden low-frequency periodicities, resulting in slowly evolving behavior, along with the short-term characteristics, makes it necessary to expand the malfunction detection study in order to include temporal convolutional networks (TCN) [55], which seem to be more suitable for this case. These concerns were partially tackled in this research via the introduction of assistive statistical post-processing of the results on the second microcontroller unit (i.e., on the NodeMCU or the LoRa32u4 board).

The compilation of programs (Arduino IDE sketches) containing the trained ANN models generated by the Edge Impulse platform, tailored for the Arduino Nano 33 BLE Sense unit, required considerable time to be executed locally on a conventional hosting computer, often exceeding 15 min. For this reason, the utilization of a second simpler, inexpensive, and faster microcontroller device (such as the LoRa32u4 module or the NodeMCU board) to perform the rest of the processing and communication tasks is an option with increased practical importance, especially during the experimentation stage of the malfunction detection process.

It must be noted that the focus of this research was more on the in situ sensing part. For this reason, further networking connectivity optimization techniques, as well as the visualization or exploitation of the original malfunction notification results, e.g., using facilitating cloud platforms, are left for future deployments toward a more commercialized version of the system being discussed.

## 5. Conclusions

This work, which benefited from the rapid growth of electronics and the pairing software, highlighted the feasibility of the steps used to deliver an innovative, compact, and low-cost system able to distinguish the diverse operating conditions of a typical agricultural water pump, including common malfunctions and anomalies. The whole approach, exploiting recently emerged but inexpensive microcontrollers, typical sensors, and radio transceivers, as well as suitable software tools, introduces a modern edge AI technique in the form of on-device intelligence, which is the most efficient approach. The deployment also includes mechanisms for generating disturbances of the pump operation, thereby facilitating the training and verification of suitable neural network models. In this regard, a neural network model was trained using data corresponding to the different scenarios of the water pump operation and was optimized so as to run on minimal in situ computing equipment. Different neural network model variants were tested. The better-performing version, utilizing accelerometric data, could diagnose the type of malfunction that had occurred with an overall accuracy rate of 93.02%. The whole system works in a decentralized manner, without high-bandwidth demands, delivering large quantities of raw data towards the cloud or increased processing power therein for central decision-making. These arrangements result in simpler infrastructure, lower energy footprints, and increased privacy, while the easy and non-invasive installation of the pump equipment is possible.

This article also facilitates future research, as it reports on the strengths and weaknesses of characteristic implementation alternatives for adding on-device intelligence to locally installed agricultural equipment. As the next steps, more optimized variants of the proposed system will be assessed in terms of the hardware selection, neural network model accuracy, standardization of the setup process, networking options, power autonomy, and user-friendliness. Finally, an upgraded version of the commercial standards will be a significant priority.

## Figures and Tables

**Table 1:** A representation of the confusion matrix for binary classification purposes.

	Actual Positive Class	Actual Negative Class
Predicted Positive Class	TP	FP
Predicted Negative Class	FN	TN

**Table 2:** The confusion matrix corresponding to the trained neural network model, created by classifying approximately 200 episodes for each class.

Class	Noise	Normal	Inlet Choke	Outlet Choke	Air Intake
Noise	200	0	0	0	0
Normal	0	190	1	0	14
Inlet choke	0	0	202	0	6
Outlet choke	0	12	2	174	15
Air intake	14	0	7	2	207

**Table 3:** The performance metrics of each class of the trained neural network model.

	Precision (%)	Recall (%)	Specificity (%)	F1-Score (%)	G-Mean1 (%)
Noise	100.00	93.46	100.00	96.62	96.67
Normal	92.68	94.06	98.22	93.37	93.37
Inlet choke	97.12	95.28	99.28	96.19	96.19
Outlet choke	85.71	98.86	96.67	91.82	92.05
Air intake	90.00	85.54	97.14	87.71	87.74

**Figure A1:**
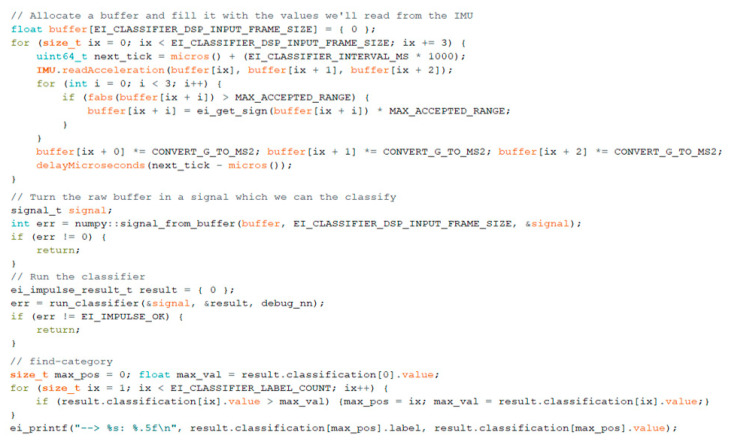
The software implementation details for the proposed system. The main parts of the code run on the ML-capable microcontroller.

## Data Availability

The data presented in this study are available upon request.

## Appendix A

The appendix provides further details on the software implementation process in order for the on-device malfunction detection part to operate fluently. Figure A1 contains the main parts of the code running on the ML-capable microcontroller for the NN model to distinguish the diverse states and potential malfunctions of the agricultural water pump.

More specifically, the Edge Impulse platform generates a library containing the trimmed-down (i.e., quantized) neural network model containing integer coefficients. This library, in addition to the trained network model, contains examples on how this model can be utilized. The proposed implementation is based on the nano_ble33_sense_accelerometer.ino example, which is easily accessible via the Examples → EdgeImpulseProjectName option, from within the Arduino IDE menu (where the “EdgeImpulseProjectName” corresponds to the name of the project, created using the Edge Impulse platform for the malfunction identification case being discussed).

Accordingly, the algorithm waits until EI_CLASSIFIER_DSP_INPUT_FRAME_SIZE values are gathered by the sensor. The accelerometric data are three-dimensional; thus, for each loop repetition, three numbers for the X, Y, and Z axes, namely buffer[ix+0], buffer[ix+1], and buffer[ix+2], are stored. When all values are stored inside the corresponding container array (buffer) and the digital pre-processing task is performed, the classification module is called and a vector is prepared, containing the matching scores for the current pump activity sample with each one of the classes or categories defined in the neural network model.

The original exemplary code (provided by the Edge Impulse platform) is modified so as to select the class with the highest matching value via the sorting process. The latter class name and its corresponding numeric identifier are printed and output via the serial port of the Arduino Nano 33 BLE Sense unit toward the NodeMCU module for delivery to the sink or gateway node.
